# Supplementary material for: Characterization of HER2-Low Breast Tumors among a Cohort of Colombian Women
Source: Cancers (Basel). 2024 Sep 12;16(18):3141. doi: 10.3390/cancers16183141 (PMC11430567; doi:10.3390/cancers16183141)
Supplement: Supplementary file 1 [file cancers-16-03141-s001.zip › Table S1.pdf]

Table S1. Differences in clinicopathological characteristics between HER2 1+ and HER2 2+ cases.

	level	HER2 1+	HER2 2+	*p* value
**n**		47	50	
**Age of diagnosis**	<50 years	12 (25.5)	8 (16.0)	0.364
	≥50 years	35 (74.5)	42 (84.0)	
**AJCC Clinical stage**	I	9 (19.1)	6 (12.0)	0.573
	II	19 (40.4)	24 (48.0)	
	III/IV	19 (40.4)	20 (40.0)	
**Scarff-Bloom Richardson**	I	7 (14.9)	6 (12.0)	0.07
	II	32 (68.1)	25 (50.0)	
	III	8 (17.0)	19 (38.0)	
**Tumor size**	≤20 mm	13 (27.7)	13 (26.0)	0.828
	21-49 mm	18 (38.3)	23 (46.0)	
	≥50 mm	14 (29.8)	13 (26.0)	
**Histological invasion**	No	20 (42.6)	20 (40.0)	0.956
	Yes	23 (48.9)	26 (52.0)	
**Lymph node involvement**	No	21 (46.7)	21 (42.0)	0.802
	Yes	24 (53.3)	29 (58.0)	
**Neoadjuvant treatment**	Received	31 (67.4)	24 (48.0)	0.087
	Did not receive	15 (32.6)	26 (52.0)	
**Type of neoadjuvant therapy**	Cytotoxic	22 (71.0)	17 (70.8)	0.632
	Hormonal	2 (6.5)	2 (8.3)	
	Cytotoxic + Hormonal	4 (12.9)	1 (4.2)	
	Cytotoxic + Trastuzumab	3 (9.7)	4 (16.7)	
**Neoadjuvant treatment response**	Complete	2 (9.5)	5 (27.8)	0.375
	Stable	3 (14.3)	2 (11.1)	
	Partial	15 (71.4)	9 (50.0)	
	Progression	1 (4.8)	2 (11.1)	
**Surgical management**	Mastectomy	22 (46.8)	27 (54.0)	0.614
	Quadrantectomy	25 (53.2)	23 (46.0)	
**Type of adjuvant therapy**	Cytotoxic	0 (0.0)	7 (14.3)	0.060
	Hormonal	27 (65.9)	26 (53.1)	
	Cytotoxic + Hormonal	11 (26.8)	10 (20.4)	
	Trastuzumab + Cytotoxic and/or Hormonal	3 (7.3)	6 (12.2)	
**Radiotherapy**	Received	40 (95.2)	43 (89.6)	0.545
	Did not receive	2 (4.8)	5 (10.4)	
**Ki67 status**	High (≥20%)	17 (36.2)	31 (62.0)	0.019
	Low (<20%)	30 (63.8)	19 (38.0)	
**ER status**	Negative	3 (6.4)	7 (14.0)	0.369
	Positive	44 (93.6)	43 (86.0)	
**PR status**	Negative	6 (12.8)	12 (24.0)	0.246

	Positive	41 (87.2)	38 (76.0)	
**Intrinsic subtype**	ER+/HER2- (luminal A-like)	26 (55.3)	0 (0.0)	<0.001
	ER+/HER2- (luminal B-like)	18 (38.3)	29 (58.0)	
	ER-/HER2-	3 (6.4)	0 (0.0)	

AJCC: American Joint Committee on Cancer; ER: estrogen receptor; PR: progesterone receptor.

* Patients with missing data or who did not receive neoadjuvant/adjuvant treatment or radiotherapy were not included in the statistical analysis.
