# Supplementary figures and images for: Characterization of HER2-Low Breast Tumors among a Cohort of Colombian Women
Source: Cancers (Basel). 2024 Sep 12;16(18):3141. doi: 10.3390/cancers16183141 (PMC11430567; doi:10.3390/cancers16183141)

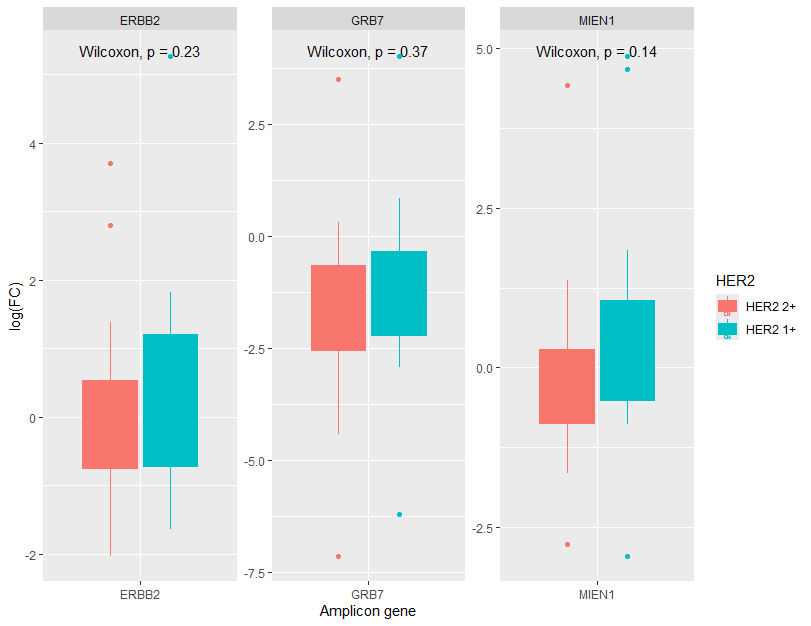

Supplement: Supplementary file 1 [file cancers-16-03141-s001.zip › Fig S1.tiff]
